# Protocol for a systematic review of outcomes from microsurgical free-tissue transfer performed on short-term collaborative surgical trips in low-income and middle-income countries

**DOI:** 10.1186/s13643-021-01797-0

**Published:** 2021-09-08

**Authors:** Henry T. de Berker, Urška Čebron, Daniel Bradley, Vinod Patel, Meklit Berhane, Fernando Almas, Gary Walton, Mekonen Eshete, Mark McGurk, Dominique Martin, Calum Honeyman

**Affiliations:** 1 Department of Burns and Plastic Surgery, Wythenshawe Hospital, Southmoor Road, Manchester, UK; 2 Department of Hand, Plastic, Reconstructive and Burn Surgery, University of Tübingen, Tübingen, Germany; 3 King’s College London, Faculty of Dentistry, Oral and Craniofacial Sciences, Guy’s Tower, Guy’s Hospital, London, UK; 4 Oral Surgery Department, Guy’s Dental Institute, Guy’s & St Thomas’ NHS Foundation Trust, London, UK; 5 Department of Plastic and Reconstructive Surgery, ALERT Hospital, Addis Ababa, Ethiopia; 6 Department of Cranio-Maxillofacial and Reconstructive Surgery, Saint Judes General Hospital, Federal Hospital of Rio de Janeiro, Rio de Janeiro, Brazil; 7 Department of Head and Neck Surgery, University Hospitals Coventry and Warwickshire, Coventry, UK; 8 Department of Surgery, School of Medicine, College of Health Sciences Addis Ababa University, Addis Ababa, Ethiopia; 9 Department of Head and Neck Surgery, University College London Hospital, London, UK; 10 Marseille, France; 11 Department of Plastic, Reconstructive and Burns Surgery, Ninewells Hospital, Dundee, Scotland, UK

**Keywords:** Free flap, Free tissue transfer, Microsurgery, Short-term collaborative surgical trips, Surgical missions, Low-income and middle-income countries, Resource limited settings

## Abstract

**Abstract:**

**Background:**

In many units around the world, microsurgical free-tissue transfer represents the gold standard for reconstruction of significant soft tissue defects following cancer, trauma or infection. However, many reconstructive units in low-income and middle-income countries (LMICs) do not yet have access to the resources, infrastructure or training required to perform any microsurgical procedures. Long-term international collaborations have been formed with annual short-term reconstructive missions conducting microsurgery. In the first instance, these provide reconstructive surgery to those who need it. In the longer-term, they offer an opportunity for teaching and the development of sustainable local services.

**Methods:**

A PRISMA-compliant systematic review and meta-analysis will be performed. A comprehensive, predetermined search strategy will be applied to the MEDLINE and Embase electronic databases from inception to August 2021. All clinical studies presenting sufficient data on free-tissue transfer performed on short-term collaborative surgical trips (STCSTs) in LMICs will be eligible for inclusion. The primary outcomes are rate of free flap failure, rate of emergency return to theatre for free flap salvage and successful salvage rate. The secondary outcomes include postoperative complications, cost effectiveness, impact on training, burden of disease, legacy and any functional or patient reported outcome measures. Screening of studies, data extraction and assessments of study quality and bias will be conducted by two authors. Individual study quality will be assessed according to the Oxford Evidence-based Medicine Scales of Evidence 2, and risk of bias using either the ‘Revised Cochrane risk of bias tool for randomized trials’ (Rob2), the ‘Risk of bias in non-randomized studies of interventions’ (ROBINS-I) tool, or the National Institute for Health Quality Assessment tool for Case Series. Overall strength of evidence will be assessed according to the Grading of Recommendations, Assessment, Development and Evaluations (GRADE) approach.

**Discussion:**

To-date the outcomes of microsurgical procedures performed on STCSTs to LMICs are largely unknown. Improved education, funding and allocation of resources are needed to support surgeons in LMICs to perform free-tissue transfer. STCSTs provide a vehicle for sustainable collaboration and training. Disseminating microsurgical skills could improve the care received by patients living with reconstructive pathology in LMICs, but this is poorly established. This study sets out a robust protocol for a systematic review designed to critically analyse outcomes.

**Systematic review registration:**

PROSPERO 225613

**Supplementary Information:**

The online version contains supplementary material available at 10.1186/s13643-021-01797-0.

## Background

Five billion people around the world are living without access to adequate surgical care [1]. Short-term collaborative surgical trips (STCSTs) provide an opportunity for surgical teams from higher income nations to collaborate with surgeons from low-income and middle-income countries (LMICs) to treat those most in need. Historically, some STCSTs have been criticised for an unsustainable ‘fly-in-fly-out’ model of surgical care delivery in LMICs, with limited patient follow-up after discharge. One consequence of this is that surgeons working in resource-limited settings have a poor context-specific evidence base to work from [2]. However, many STCSTs return annually to the same centre, building lasting relationships, offering sustainable education and training and treating patients who would otherwise have no access to healthcare. This model offers greater opportunity for thorough follow-up. Indeed, new methods of long-term patient follow-up after discharge are emerging that aim to improve patient safety after STCSTs in LMICs [3, 4]. Historically, such trips were often referred to as ‘missions’. However, there is a growing consensus that the model described above is better captured by the term ‘collaboration’ [5].

Free-tissue transfer can be considered the gold-standard method of reconstruction after significant composite tissue defects from cancer, trauma and infections [6–9]. In the largest series from high volume centres, flap failure rates as low as 0.6%, and take back rates of 1.5% (66% successful salvage rate) have been published [10]. For surgeons operating in LMICs, that do not have funding and access to the required equipment and training to facilitate independent microsurgical practice, treatment options are restricted to those used 50 years ago in countries that now perform regular free-tissue transfer. For select advanced pathology, this may be the difference between amputation and lower limb salvage after trauma and sarcoma resection, or poorer functional outcomes following head and neck cancer reconstruction [11–15]. Performing microsurgery in LMICs is challenging, even for experienced teams. However, a select few centres in LMICs are undertaking these procedures as part of standard practice [16–18]. Local and regional reconstructive alternatives do exist (e.g. transposition or pedicled flaps); however, many of these are also technically demanding, and even with extensive training, microsurgical equipment and techniques, they are vulnerable to complications requiring further surgery [19–22]. However, there is also some data in support of successful local reconstructive alternatives being used effectively in low-resource contexts [22].

There are existing reviews of STCSTs, but none provide the reader with a detailed account of microsurgical free-tissue transfer performed on this basis, and many combine surgical trips with medical ones in their analysis. There have been studies on socioeconomic/political impact, cost-effectiveness, sustainability and quality of follow-up [2, 23–25]. However, it has been difficult to draw any firm conclusions due to the paucity of high-quality quantitative data, and the heterogeneity of these interventions, which are often organised on a departmental basis [2]. The closest review to our proposed study assessed a number of outcomes from ‘short-term reconstructive missions’, although the majority of patients in their analysis underwent cleft surgery (without microsurgical free tissue transfer) [26]. They cite substantial reporting bias when it comes to operative complications, and highly variable estimates of cost effectiveness for the same intervention depending on local context.

There are some fundamental challenges to providing microsurgery in LMICs. Amongst those reported are a lack of specialist equipment, trained staff and appropriate level three post-operative monitoring facilities [27]. STCSTs, which can provide additional staff and resources, can directly address these challenges. However, these are usually short-term interventions, and patients may not be afforded the same degree of follow-up provided by the home institutions of the visiting surgeons. In addition, it is traditionally held that reconstructive surgeons should stick to ‘simple’ surgery on STCSTs [28]. Therefore, it is important to establish the safety of delivering free-tissue transfer on STCSTs. Identification of common complications and constraints will inform organisations engaged in STCSTs where to focus their training and fundraising efforts in order to improve patient outcomes. The highly specialised nature of this surgery means that it is difficult to draw on the existing review articles in the literature to make any firm conclusions about this technique performed during STCSTs.

The aim of this study is to publish a robust systematic review protocol to establish the safety and efficacy of microsurgical free tissue transfer performed on STCSTs in low-income and middle-income countries. A meta-analysis of key outcomes will be undertaken with the aim of developing potentially life-changing microsurgical practice in resource-limited settings.

## Methods

This protocol is registered on the PROSPERO international prospective register of systematic reviews (submitted 15/12/20 ID: 225613) and is written in accordance with the Preferred Reporting Items for Systematic Reviews and Meta-Analysis Protocols (PRISMA-P) guidelines [29, 30]. The methodology applied to the final systematic review and meta-analysis is derived from, and in line with, the Cochrane Handbook for Systematic Review of Interventions [31] and the Preferred Reporting Items for Systematic Reviews and Meta-Analyses (PRISMA) statement [32].

### Search strategy

The search strategy (Table 1) will be applied to the MEDLINE and Ovid EMBASE databases from inception to 5 August 2021.

**Table 1:** Electronic database search—MEDLINE and Ovid EMBASE

	Concept 1—Surgery	AND	Concept 2—Micro	AND	Concept 3—Collaboration
**MeSH term**	**Surgery**		**Free Tissue Flaps**		**Medical Missions**
**OR**			**Microsurgery**		
		**OR**	Flap*	**OR**	Collab*
		**OR**	Free Flap*	**OR**	Mission*
		**OR**	Free Tissue Transfer	**OR**	Humanitarian
				**OR**	Visit*
				**OR**	Charit*
				**OR**	Trip
				**OR**	Outreach
				**OR**	Volunt*
				**OR**	Non-government*
				**OR**	Safari
				**OR**	Blitz
				**OR**	Camp

### Study selection criteria

#### Inclusion criteria

All clinical studies in the English language reporting outcomes of any microsurgical procedure performed on a short-term reconstructive trip to any low-income or middle-income country (in accordance with the World Bank Classification [33]) will be eligible for inclusion. Studies that match the inclusion criteria performed in low-resource environments in a high-income country will also be included. Children and adults will be considered. All cases performed using either operating microscope or loupe magnification will be included, as both are successfully reported in resource-limited settings [16]. The Population, Intervention, Comparison, Outcome (PICO) model was used to determine study selection criteria (Table 2) [34].

**Table 2:** Population, Intervention, Comparison, Outcome (PICO)

Population	Intervention	Comparator	Outcomes
Patients operated on during STSCs	Reconstructive surgery by microvascular free-tissue transfer	Data from the UK National Flap Registry [9]	Rate of complications
In low- and middle-income countries or low-resource health environments	Performed during a visiting surgical mission		Nature of complications
Children and adults to be included	This will include both loupes and microscope magnification		Rate of return to theatre
			Rate of free flap salvage
			Nature of attempted salvage
			Measures of cost-effectiveness
			Assessments of impact on local training
			Impact on burden of disease/waiting list
			Long-term ‘legacy’—e.g. establishment of a local microsurgery unit or fellowship from host to visiting institution

#### Exclusion criteria

Studies that do not provide sufficient data for comparative analysis will be excluded. Where incomplete or absent data is presented in a given study, study authors will be contacted by email on a maximum of two separate occasions, 2 weeks apart, inviting them to provide further data before being excluded. Data presented from microsurgical units already independently performing microsurgery in low-income or middle-income countries, not on a STCST, will also be excluded.

#### Study design

Randomised controlled trials (RCTs), cohort, case-control and case series will be considered for inclusion. Pilot searches indicate that the majority of studies will be case series of varying sizes. As such, no limitation regarding study size or clinical follow-up will be made. Case reports, letters, opinion pieces and literature reviews will be excluded. Any unpublished or ongoing prospective clinical trials will also be excluded.

#### Participants

The participants are children or adults that have undergone microsurgical free-tissue transfer during a short-term collaborative surgical trip to a low-income or middle-income country. No limitations based on patient demographics, body region or aetiology will be imposed.

### Outcomes

#### Primary outcomes

The primary outcomes are rate of free-flap failure and rate of emergency return to theatre for attempted free-flap salvage. Return to theatre will be classified as a donor site or anastomotic complication. The time taken to return to theatre, and the rate of successful flap salvage will also be documented where available. Where disclosed, we will record the method of flap salvage attempted.

#### Secondary outcomes

The secondary outcomes will include complications and any functional or patient-reported outcome measures included. Based on previous large case studies of free flaps, complications will be divided into medical and surgical. The surgical group will be further subdivided into intraoperative and post-operative. Complications will be stratified according to the Clavien-Dindo classification [35–37]. Finally, if available any assessment of pre-operative fitness will be recorded.

#### Additional data

In addition to the primary and secondary outcomes, duration of procedure and length of stay will also be recorded where available. In the context of a STCST to a LMIC, this is particularly important. The following will also be extracted: bibliographic data (title, author, date), study characteristics (design, method of randomisation/allocation, blinding, number of participants, groups/subgroups), mission characteristics (country, length of mission, type of mission/subspecialty, organisation (type and size), type of hospital (e.g. public or private), frequency of missions), patient characteristics (age, sex, indication for surgery, comorbidities, smoking status), intervention characteristics (operation(s) performed, duration of operation, length of stay, who performed the surgery (local or visiting surgeon), experience level of surgeon who performed anastomosis and raised flap, pre-operative workup), and rate and duration of follow-up.

Outcomes will be compared to data available from the multicentre, UK National Flap Registry, published in 2019 [9].

#### Data management and extraction

Abstracts will be screened on the Rayyan systematic review software tool. Full papers will be downloaded as PDFs, and stored locally on Mendeley Desktop. All abstracts included will proceed to full-text analysis unless it is immediately apparent following reading of the introduction that they are irrelevant. Data items will be collected in a standardised data collection proforma. For instances of incomplete data, we will contact the corresponding author. If 2 weeks elapse with no response, we will repeat this request once.

### Data selection

Screening will be conducted and recorded in accordance with the Preferred Reporting Items for Systematic Review and Meta-Analysis (PRISMA) guidelines [32]. Abstracts screened according to criteria set out in this protocol by two independent researchers (HdB and UC). In case of disagreements, researchers will meet to discuss disparities; if there are still disagreements, a third author (CH) will make the final decision on inclusion. If abstracts are not available on assessment, the paper will be downloaded in full for analysis.

### Risk of bias assessment

Each study will be assessed for risk of bias by two independent reviewers (HdB and UC) according to an appropriate validated tool. Randomised studies will be assessed using the revised Cochrane risk of bias tool for randomised trials (Rob2) [38]. Non-randomised studies will be assessed using the ‘Risk of bias in non-randomised studies of interventions’ (ROBINS-I) tool [39]. Case series will be assessed using the National Institute of Health Quality Assessment Tool for Case Series Studies [40].

### Quality of studies

Each study will be assessed according to the Oxford Evidence-based Medicine Scales of Evidence 2 [41]. This data will be tabulated.

### Strategy for data analysis and synthesis

Statistical analysis of included studies will be undertaken in R version 4.0.5 (R Foundation for Statistical Computing, Vienna, Austria). Patient demographics will be presented using basic descriptive statistics. Complications, free flap failure, emergency return to theatre and successful free-flap salvage will be calculated and displayed as rate (%). Data from the first UK National flap registry will be used as a comparator [9]. Statistical heterogeneity will be examined by calculating *I*^2^ and Cochran’s *Q* statistic, and interpreted according to Cochrane guidance on determining heterogeneity [31]. If *I*^2^ > 50, a random-effects model (DerSimonian and Laird with a logit transformation applied) will be used to calculate relative risk with 95% confidence intervals [42]. If *I*^2^ ≤ 50, a fixed-effects model will be used for relative risk calculations. A *p* value of < 0.05 will be considered statistically significant. The results of this meta-analysis will be presented in Forest plots. Funnel plots will be used to detect publication bias. If quantitative analysis is inappropriate, a narrative synthesis will be performed.

The analysis detailed above will be undertaken for the pooled data. If sufficient data is available, subgroup analysis by region (e.g. head and neck, trunk etc.) will be undertaken.

### Confidence in cumulative evidence

The body of evidence underpinning each of the findings will be assessed according to the ‘Grading of Recommendations, Assessment, Development and Evaluations’ (GRADE) approach [43]. Using this approach, the authors will express their ‘certainty’ that the body of evidence reflects reality (high, moderate, low, very low).

## Discussion

This will be the first systematic review and meta-analysis of the outcomes of free-tissue transfer performed on STCSTs to low-income and middle-income countries. Our pilot searches indicate that the majority of data will be published in case series’; it is difficult to form a conclusion from any one of these alone. This underlines the importance of a thorough synthesis of these studies into a unifying review article. However, lack of randomised allocation and control groups increase the risk of bias, and this has led to concerns regarding the quality of evidence in this field [26]. We cannot remedy this, but will provide the reader with an open appraisal of the body of evidence according to the GRADE approach. From this, they will be able to form their own conclusions about their confidence in our findings.

We predict considerable clinical heterogeneity between the studies included in this paper. STCSTs are often based on relationships between specific institutions, sometimes individual surgeons, and thus there is no ‘one size fits all approach’. Another source of heterogeneity will come from the inclusion of all flap types. We will try to mitigate this through sub-group analysis if possible, but there may be insufficient data available.

Nutritional status has been identified as a determinant of free flap outcome [44]. STCSTs treat a diverse population of patients, many of whom will have poor nutritional status. This may be a confounding factor in reconstructive outcomes, but is unlikely to be well documented in a standardised manner across the literature. We will collect nutritional and other preoperative assessments where possible, but there may not be sufficient data to adjust for these variables.

We hope to equip surgeons undertaking STCSTs with the relevant data to offer appropriate treatment to their patients in order to achieve the best possible outcomes. In addition, this will ensure patients are able to give informed consent with an understanding of the risks specific to their situation. These impacts will improve patient safety, reconstructive outcomes and follow-up.

Finally, with appropriately strict governance surrounding distribution of funds designed to provide healthcare in low- and middle-income countries, it is essential that actors in this field are able to provide evidence supporting their work. Through identifying the nature and severity of complications, we hope to inform surgeons and funders of the challenges and barriers to free tissue transfer in LMICs. This should encourage investment in areas that are most likely to improve patient outcomes. This study will assist those funding global surgery to allocate resources to appropriate interventions with proven patient benefit.

## Supplementary Information

**Additional file 1.** PRISMA-P Checklist.

## Data Availability

Data sharing is not applicable to this article as no datasets were generated or analysed during the current study.

## Abbreviations

EMBASE: Elsevier Biomedical and Pharmacological Bibliographic Database
GRADE: Grading of Recommendations, Assessment, Development and Evaluations
LMICs: Low- and middle-income countries
MEDLINE: US National Library of Medicine Bibliographic Database
MeSH: Medical Subject Headings
PRISMA: Preferred Reporting Items for Systematic Reviews and Meta-Analyses
PROSPERO: International Prospective Register of Systematic Reviews
RCT: Randomised controlled trial
Rob2: Revised Cochrane risk of bias tool for randomised trials
Robins-I: Risk of bias in non-randomised studies of interventions
STCST: Short-term collaborative surgical trip
